# 3D-Printed Microdevices: From Design to Applications

**DOI:** 10.3390/mi15060791

**Published:** 2024-06-15

**Authors:** Cristiane Kalinke, Rodrigo A. A. Muñoz

**Affiliations:** 1 Institute of Chemistry, University of Campinas, Campinas 13083-970, SP, Brazil; 2 Department of Chemistry, Federal University of Paraná, Curitiba 81531-980, PR, Brazil; munoz@ufu.br

3D printing represents an emerging technology in several fields, including engineering, medicine, and chemistry. The manufacturing of miniaturized devices is becoming increasingly popular among users pursuing the homemade fabrication of reproducible and low-cost microdevices in a single step [1]. It is important to highlight that the miniaturization of devices also leads to a reduction in the amount of printing materials used and the generation of waste [2]. Additionally, 3D printing can provide advantages to the fabrication of these devices, including reduced production times and the customization of materials, sizes, and shapes [3]. High resolution and printing quality can also be achieved by using 3D printing, allowing the fabrication of small parts, microchannels for microfluidic systems, electrical contacts and circuits, sensors and complete miniaturized devices [4,5]. Such features also contribute to the interconnection and automation of systems, allowing the development of novel and portable complete devices, such as lab-on-a-chip approaches [6,7]. Parallel to this, the combination with Internet of Things (IoT) approaches is another advantage because it facilitates real-time monitoring [8,9,10].

In other words, 3D printing creates thousands of possibilities for the development of miniaturized and portable devices for the most varied applications, from academic research to industrial areas. This can be attributed to the popularity of 3D printing methods and printers, in addition to the low cost of equipment and the easy and fast prototyping it enables [11]. In this context, the Special Issue entitled “3D-Printed Microdevices: From Design to Applications” showcases distinct microfluidic, miniaturized and microgravity devices and systems manufactured using 3D printing to be applied in different fields such as engineering, analytical, electrochemical, biochemical, biological and medical applications.

For example, the first study authored by Kortmann et al. reports the prototyping of a system for the purification of antibodies based on continuous chromatography. This method enables continuous separation processes using a miniaturized 3D-printed device, producing lower-cost monoclonal antibodies and using fewer materials compared to the traditional method. The device was printed using an acrylate material followed by UV curing, and hydroxylated wax was used as the supporting material. In addition, UV photometers used for protein quantification were also manufactured by 3D printing using different printers and materials. The system showed versatile design customization, fast prototyping, improved space–time yield, and it was tested for use with different chromatographic techniques and units, demonstrating potential to be applied for the purification of products by bind-and-elute separation techniques.

As has been noted, 3D printing has been widely employed for the manufacture of flow-based systems, in which stereolithography (SLA) stands out, enabling a high resolution for the printing of small-sized flow channels. In this regard, Vedhanayagam et al. developed microfluidic channels (<100 µm) by digital-light projection (DLP) printing using a photosensitive resin and polydimethylsiloxane (PDMS) molds, followed by UV light curing. The printed parts were subjected to a sequence of post-treatments to remove uncured resin, eliminate photoinitiators, enhance PDMS curing and release, and avoid mold warping. Different 3D-printed designs were proposed, demonstrating the versatility of 3D printing for the development of droplet microfluidics devices. The authors also proposed a method for recycling the solvent (isopropyl alcohol) used during the ultrasonication post-treatment process, which needs to be encouraged to reduce solvent waste and costs. 

In the next study, Awate et al. designed and compared two different configurations of flow-focusing devices, which allowed the SLA printing of mili- or microfluidic channel devices. For the second device, the mold was also produced by SLA followed by its encapsulation with PDMS. The devices were evaluated according to the sheath flow rate and flow-focusing width effects. The authors demonstrated that the microfluidic-based device showed better hydrodynamic focusing, which could be attributed to its geometry. Furthermore, it is possible to highlight that no post-treatment of the parts was necessary after printing, which corroborates with the fast and low-cost printing process for the manufacture of devices.

In another approach, a 3D-printed ink film based on a piezoelectric direct drive was reported by Liu et al. The electrode was produced by printing and curing a silicon dioxide ink film on the substrate using a piezoelectric ceramic driver. Printing parameters were compared and optimized to enhance the film printing. In this case, microjet droplets allowed control of the thickness of the printed layers and the film, and printing stability was achieved. Circular film electrodes were applied as thermal batteries, showing stable electrical performance. Furthermore, the device stood out due to its easy operation and fast and precise response.

Chan et al. fabricated an automated sample collection, preparation and analysis device from modified 3D printers. Reagent wells and cartridges were 3D printed via stereolithography. The most impressive innovation of this study is that the device, well plates, and cartridges were tested and shown to work in a microgravity environment, suggesting that the system is able to be used in the International Space Station. The device was tested with different samples (i.e., water and biological samples, among others), allowing the extraction, purification, and detection of genetic materials (RNA and DNA) for future applications, for example, in the health monitoring of workers in space. 

The last article in this Special Issue is authored by Cataño et al. and reported on the development of a 3D-printed programmable microfluidic peristaltic pump with a low manufacturing cost (~USD 175). For this, the pump parts were 3D printed by DLP using a polycarbonate-based resin. A microfluidic vasculature model was bioprinted, and a bioreactor was also 3D printed by DLP using polycarbonate resin. Additionally, the system can be used for multiplexed tests and supports high humidity, which is desirable as it facilitates its application in perfusion cell culture for organ-on-a-chip devices. 

As has been noted, 3D printing has been employed on different fronts, enabling the prototyping and development of microdevices. Three-dimensional printers are easy to operate and facilitate fast printing and high resolution in the fabrication of complex and small-sized parts. Furthermore, the possibility of building alternative devices to other expensive systems is another advantage of 3D printing. Thus, new and improved devices can be easily built via 3D printing using cheaper materials and equipment. Considering the applications of microdevices, different 3D printed approaches are highlighted, including microfluidic, chromatographic, battery, and organ-on-a-chip devices. This demonstrates the versatility of using this outstanding technology in different fields.
